# Plant Growth Promotion by Two Volatile Organic Compounds Emitted From the Fungus *Cladosporium halotolerans* NGPF1

**DOI:** 10.3389/fpls.2021.794349

**Published:** 2021-12-03

**Authors:** Lingmin Jiang, Myoung Hui Lee, Cha Young Kim, Suk Weon Kim, Pyoung Il Kim, Sung Ran Min, Jiyoung Lee

**Affiliations:** ^1^Biological Resource Center, Korea Research Institute of Bioscience and Biotechnology, Jeongeup, South Korea; ^2^Wheat Research team, National Institute of Crop Science, Rural Development Administration, Wanju, South Korea; ^3^Center for Industrialization of Agricultural and Livestock Microorganisms (CIALM), Jeongeup, South Korea; ^4^Plant Systems Engineering Research Center, Korea Research Institute of Bioscience and Biotechnology, Daejeon, South Korea

**Keywords:** volatile organic compounds, *Cladosporium* sp. strain, plant growth promotion, 2-methyl-butanal, 3-methyl-butanal

## Abstract

Microbial volatiles have beneficial roles in the agricultural ecological system, enhancing plant growth and inducing systemic resistance against plant pathogens without being hazardous to the environment. The interactions of plant and fungal volatiles have been extensively studied, but there is limited research specifically elucidating the effects of distinct volatile organic compounds (VOCs) on plant growth promotion. The current study was conducted to investigate the impact of VOCs from *Cladosporium halotolerans* NGPF1 on plant growth, and to elucidate the mechanisms for the plant growth-promoting (PGP) activity of these VOCs. The VOCs from *C. halotolerans* NGPF1 significantly promoted plant growth compared with the control, and this PGP activity of the VOCs was culture medium-dependent. Headspace solid-phase microextraction (HS-SPME) coupled with gas chromatography–mass spectrometry (GC–MS) identified two VOC structures with profiles that differed depending on the culture medium. The two compounds that were only produced in potato dextrose (PD) medium were identified as 2-methyl-butanal and 3-methyl-butanal, and both modulated plant growth promotion and root system development. The PGP effects of the identified synthetic compounds were analyzed individually and in blends using *N. benthamiana* plants. A blend of the two VOCs enhanced growth promotion and root system development compared with the individual compounds. Furthermore, real-time PCR revealed markedly increased expression of genes involved in auxin, expansin, and gibberellin biosynthesis and metabolism in plant leaves exposed to the two volatile blends, while cytokinin and ethylene expression levels were decreased or similar in comparison with the control. These findings demonstrate that naturally occurring fungal VOCs can induce plant growth promotion and provide new insights into the mechanism of PGP activity. The application of stimulatory volatiles for growth enhancement could be used in the agricultural industry to increase crop yield.

## Introduction

Volatile organic compounds (VOCs) released by microorganisms without direct contact are a major source of secondary metabolites and are used as novel signaling molecules (Schulz-Bohm et al., 2017a,b). VOCs significantly impact the fitness and dispersal of microorganisms in the ecosystem, as well as providing protection from external stimulus and promoting survival (Kanchiswamy et al., 2015; Silva et al., 2018; Hammerbacher et al., 2019; Syed-Ab-Rahman et al., 2019; Silva Dias et al., 2021). The low molecular weight and high vapor pressure of these secondary metabolites allow the diffusion of complex mixtures of solids, liquids, and gases in the aboveground and subterranean plant compartments (Schulz and Hotling, 2015; Massalha et al., 2017). VOCs also act as signaling compounds between organisms and other kingdoms, including in plant–microbial interactions and animal–microbial interactions (Hernandez-Calderon et al., 2018; Sharifi and Ryu, 2018a,b; Kaushal, 2019; Chatterjee et al., 2020; Montejano-Ramirez et al., 2020; Morcillo et al., 2020; Abreo et al., 2021; Cortes-Patino et al., 2021; Kong et al., 2021).

Fungi, as a fundamental part of the plant microbiome, comprise 2.2 to 3.8 million species (Hawksworth and Lucking, 2017), emit a large number of VOCs, and play an important role in the crosstalk of network interactions between both organisms (Wheatley, 2002; Deetae et al., 2009; Laureys and De Vuyst, 2014; Cheng et al., 2016; Ana et al., 2020; Abreo et al., 2021; Baazeem et al., 2021). The VOCs are uniquely associated with fungal metabolism, with fungi producing a cocktail of dozens to hundreds of different VOCs, including alcohols, aldehydes, acids, ethers, esters, ketones, hydrocarbons, terpene, and sulfur compounds (Korpi, et al., 2009). Among these VOCs, some compounds emitted by fungi can modify plant architecture and growth, such as sesquiterpenes (SQTs) (Ditengou et al., 2015; Zhang et al., 2020). The fungi *Trichoderma atroviride* produces 6-pentyl-2H-pyran-2-one (6-PP), which was reported to regulate root architecture in lateral root development (Kwon et al., 2010; Garnica-Vergara et al., 2016). In the same year, Kottb and co-workers obtained the opposite results, in that the 6-PP produced by fungi *Trichoderma atroviride* strain IsmT5 reduced fresh weight, root length, and leaf area in *Arabidopsis thaliana*, but enhanced defense-related compounds and related gene expression in this plant species (Kottb et al., 2015). Many studies have reported that VOCs are species-specific, and although there are numerous excellent reviews classifying the functions of these VOCs in interspecies communication (Junker and Tholl, 2013; Audrain et al., 2015; Inamdar et al., 2020; Jeong et al., 2021), the functions and biosynthetic pathways of VOCs emitted by fungi remain poorly characterized. Recognition of VOCs by receiver organisms such as plants stimulates a wide spectrum of responses, but how these VOC-mediated signals trigger responses remains a growing area of research. Bacteria are reported to promote plant growth mainly through regulation of plant hormones including auxins, cytokinins, ethylene, gibberellin, and abscisic acid, while expansins are proteins that promote cell wall extension and stimulate plant growth (Bailly and Weisskopf, 2012; Kurepin et al., 2014; Yang et al., 2014; Navarro-Leon et al., 2016; Verma et al., 2016; Tahir et al., 2017; Luo et al., 2019; Zhang et al., 2019; Bakaeva et al., 2020; Keswani et al., 2020; Cantabella et al., 2021). However, for fungi, research on the interactions of VOCs and plant hormone-regulated pathways is still in the preliminary stages. It is currently suggested that VOCs promote plant growth and induce defense pathways *via* processes dependent on metabolome or/and proteome alterations, and these processes are closely linked to auxin signaling or phytohormone signaling pathways, such as JA (jasmonic acid) and SA (salicylic acid) pathways. Many fungal species and strains including *Cladosporium cladosporioides* CL-1 (Paul and Park, 2013), *Phoma* sp. GS8-3 (Naznin et al., 2013), and *Trichoderma atroviride* (Garnica-Vergara et al., 2016; Lee et al., 2016) were shown to promote plant growth through auxin pathways. Zhang and colleagues reported that VOCs emitted by *Bacillus subtilis* GBO3 increased the auxin level in *Arabidopsis*, which initiated plant growth promotion (Zhang et al., 2007). Furthermore, ethylene biosynthesis-related genes, *ACO-1*, *ACS4*, *ACS12*, and *SAM-2*, were downregulated, as mediated by JA and SA signaling pathways. Increased expression levels of genes encoding the expansin, auxin, gibberellin, and cytokinin, and ethylene-related genes were also observed in response to VOCs from *B. subtilis* SYST2 (Tahir et al., 2017). However, additional details, such as whether multiple signaling pathways are involved in the growth-promoting response of VOCs or whether multiple signals converge on a common pathway, are yet to be elucidated.

The current study aimed to identify efficient volatiles emitted by *Cladosporium halotolerans* NGPF1 and characterize their role in crosstalk with plants. The growth-promoting activity of these VOCs, including in plants and medium, was explored. In addition, differential gene-expression patterns were investigated to identify genes that could potentially explain the key points of how the VOCs promote plant growth. The findings from this study are of particular significance for the agricultural application of VOCs to promote plant growth.

## Materials and Methods

### Fungal Isolation, Identification, and Culture Media

Fungal strain *Cladosporium halotolerans* NGPF1 was isolated from contaminated media of *Nicotiana benthamiana* seedlings in the Korea Research Institute of Bioscience and Biotechnology (KRIBB), Daejeon, South Korea (36°22′38″ N, 127°21′35″ E). Identification of the fungus at the species level was conducted by BioFact Inc. (Daejeon, Korea) through amplification of the 5.8S rRNA using ITS1/ITS4 primers. Phylogenetic trees were reconstructed in MEGA (ver. 7) using the neighbor-joining (NJ) method. The purified isolate was precultured from frozen 15% glycerol stocks on potato dextrose agar (PDA, Difco, USA) for 3days at 25°C and routinely cultured on PDA. The fungal NGPF1 strain was deposited at the Korean Collection for Type Cultures (KCTC; https://biorp.kribb.re.kr) as bio-product BP1429732. The media PDA, MEA (malt extract, Difco), M9A (Difco), R2A (Difco), LB (Difco) and Murashige and Skoog (MS salt, Duchefa, Netherlands) were all used in this study.

### Plant Material, Growth Condition and Soil Experiments

*Nicotiana benthamiana*, tomato (Micro-Tom), kimchi cabbage, bok choy, broccoli, and carrot seeds were surface sterilized (70% ethanol for 5min, 2.5% sodium hypochlorite for 15min) and rinsed three times with sterile distilled water for 5min (Jiang et al., 2019). All sterilized plant seeds were sown on half-strength MS medium and plants were grown in a growth chamber at 25°C and 55–60% relative humidity (RH) with a 16-h light/8-h dark cycle.

To prepare fungal spore suspensions, a 7-day-old culture of *C. halotolerans* NGPF1 was collected in sterile distilled water and filtered through eight layers of cotton gauze. After adjusting the density to 10^8^ spores/mL, the suspension was spotted on half of I-plates except where otherwise indicated. For investigation of the effects of fungal VOC on vegetable plants, *C. halotolerans* NGPF1 was pre-inoculated on PDA medium on one side of the split plate for 7days, and four surface-sterilized vegetable plant seeds (kimchi cabbage, cabbage, broccoli, and carrot) were germinated on MS agar medium on the other side of the plate at 25±1°C for 4days. Six-day-old tomato and *N. benthamiana* seedlings were co-cultured with *C. halotolerans* NGPF1 for 10days.

For the soil experiment, plates inoculated with *C. halotolerans* NGPF1 were placed in 15-L plastic boxes and plant seeds (bok choy and kimchi cabbage) were sown into a 100×40mm culture dish containing autoclaved soil, and then moved into a growth chamber at 25±1°C with a 16-h light/8-h dark photoperiod for 10days.

### Plant Growth Quantification

To test the PGP activity of VOCs from *C. halotolerans* NGPF1 grown on different media, a modified plate-within-a-plate method was used. Briefly, *N. benthamiana* seeds were germinated on MS medium containing 3% sucrose and 0.4% gelrite (Duchefa, Netherlands). Simultaneously, 0.5-cm agar blocks of *C. halotolerans* NGPF1 grown for 7days were placed on PDA, MEA, M9, R2A, LB, and MS agar media in 35×10mm petri dishes, and the petri dishes were placed into a culture dish (100×40mm) to permit volatile exchange. The culture dishes were incubated in a growth chamber at 25°C with a 16-h light/8-h dark photoperiod. After the specified number of days, the whole aerial part and root part of the plants were separated and evaluated in terms of leaf number, lateral root number, leaf fresh weight, root length, and fresh weight. Total chlorophyll contents were calculated as described by Fu et al. (2016). Experiments were repeated at least twice with similar results, and the data were analyzed statistically.

### Gas Chromatography–Mass Spectrometry (GC–MS)

*Cladosporium halotolerans* NGPF1 was grown in 200ml PD broth or MS broth in 500-mL flasks at 25°C for 7days, then the hyphae were collected and freeze-dried. Headspace VOCs were collected at 100°C for 60min. GC–MS analysis was performed *via* Agillent 5975C GC/MSD, with a flow rate of 1ml/min helium gas. The SPME fibers were desorbed at 50°C for 10min, and 10°C/min rate increased to 250°C, holding for 10min. GC–MS was running for 28min. The volatile compounds were analyzed based on NIST/EPA/NIH Mass Spectrum Library (V. 05).

### Effect of VOCs on Plant Growth Promotion

Different concentrations of 2-methyl-butanal (0.1μM, 1μM, and 5μM), 3-methyl-butanal (0.1μM, 1μM, and 5μM), and mixtures of the two compounds (0.01μM, 0.1μM, and 1μM of each of 2-methyl-butanal and 3-methyl-butanal) were analyzed for their effect of plant growth using the modified plate-within-a-plate method described above. Plant growth was assessed using the parameters of plant biomass and chlorophyll contents.

### RNA Extraction, cDNA Synthesis and Real-Time PCR Analyses

To explore the possible mechanisms underlying VOC promotion of plant growth, 6-day-old Micro-Tom tomato seedlings were exposed to a blend of VOCs (1μM of 2-methyl-butanal and 1μM of 3-methyl-butanal) for 5, 7, 9, and 10days. Shoots were harvested from VOC-treated or non-VOC-treated tomato plants for analysis of expression of selected genes. Total RNA was extracted from the samples using a RNAqueous™ phenol-free RNA isolation kit (Invitrogen) according to the manufacturer’s instructions, and quantity and quality of the RNA were measured by PicoGreen and Nanodrop 2000 (Thermo Scientific). First-strand cDNA was synthesized *via* a high-capacity cDNA Reverse Transcription Kit (Applied Biosystems) using random hexamer primers. To examine the relative expression level of the phytohormone-related genes as well as cell wall extension such as those encoding gibberellin (GA20ox-1), ethylene (ACO1), cytokinin (SICKX1), expansin (*EXP9*, *EXP18*), and auxin (*SIAA1*, *SIAA3*) after exposure of the plants to the VOCs, primers were designed according to the study of Tahir et al. (2017). Actin4 (*ACT4*) was used as an internal control (Lee et al., 2020).

Real-time PCR was performed using the SYBR Green Master Kit (BioFACT, Korea), which emits fluorescence to cDNA that can then be detected. Each sample was assayed in triplicate. The Bio-Rad CFX connects Real-time system was used for analysis with the following program: initial denaturation at 95°C for 15min, followed by 40cycles of denaturation at 95°C for 20s, annealing at 58°C for 20s, and extension at 72°C for 20s. Finally, the relative expression level of each gene was calculated based on the 2^-∆∆CT^ method (Livak and Schmittgen, 2001).

## Results

### Molecular Identification and Phylogenetic Analysis

**Figure 1:**
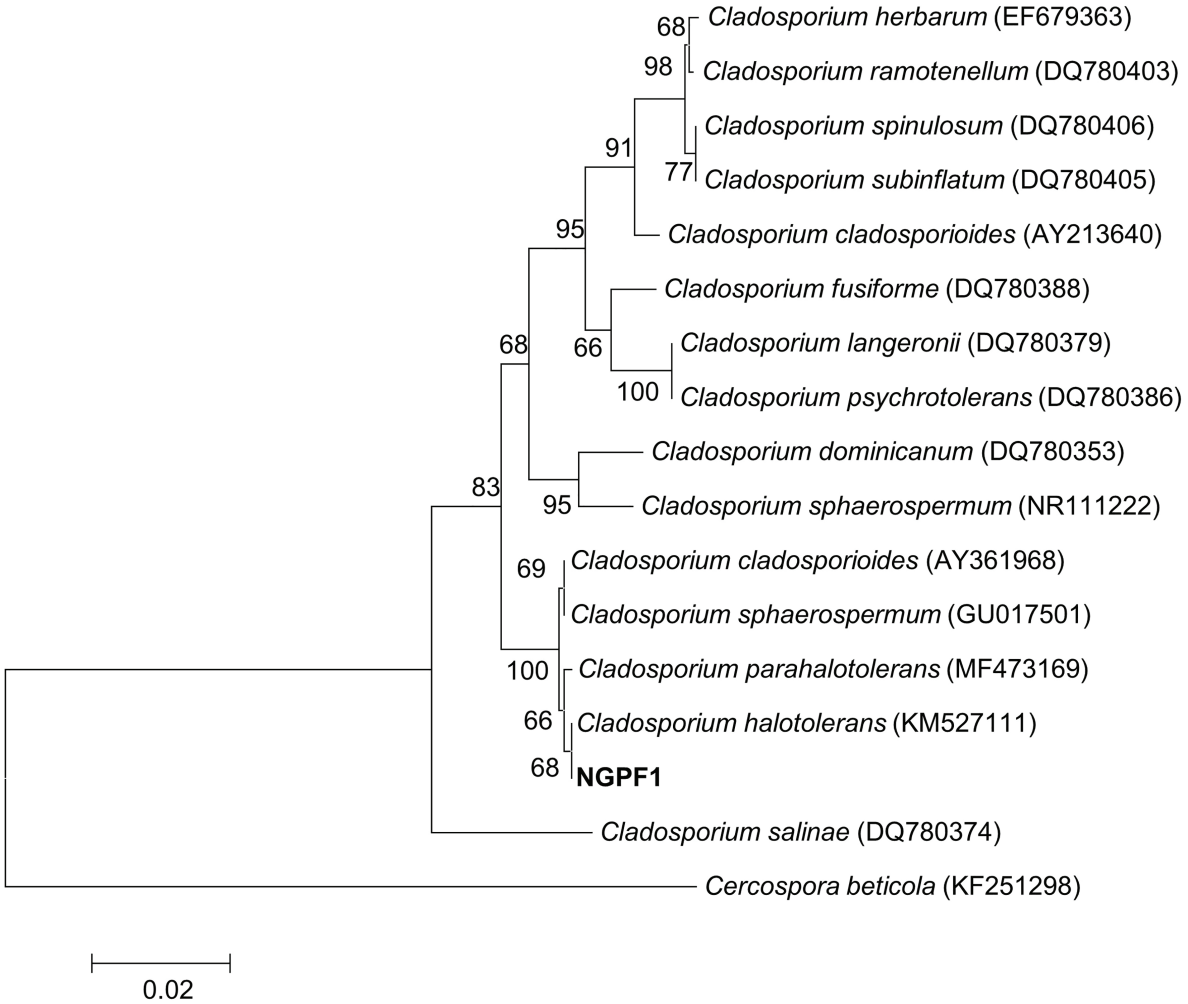
Neighbor-joining (NJ) phylogenetic tree based on ITS gene of strain NGPF1 and closely related members of the genus *Cladosporium*. Bootstrap values (>50%) calculated using the NJ algorithms are shown. Scale bar: 0.020 substitutions per nucleotide position.

A dark greenish fungus, strain NGPF1, was isolated from a contaminated media of *N. benthamiana* seedlings. The fungal strain was identified by sequencing the 5.8S rRNA region using nucleotide DNA as a template and universal primers ITS1 and ITS4. The almost full-length region of the 5.8S rRNA was used for a BLAST search of the GenBank database of the National Center for Biotechnology Information. The BLAST search revealed that strain NGPF1 exhibited the highest gene sequence similarity with the genus *Cladosporium*. Phylogenetic analysis indicated that the obtained sequence shared 100% identity with the sequence of *Cladosporium halotolerans* BCRC FU30267 (GenBank accession number KM527111), and formed a cluster with this strain (Figure 1).

### Effects of *C. halotolerans* NGPF1 VOCs on Vegetable Plant Growth *in vitro* and *in vivo*

**Figure 2:**
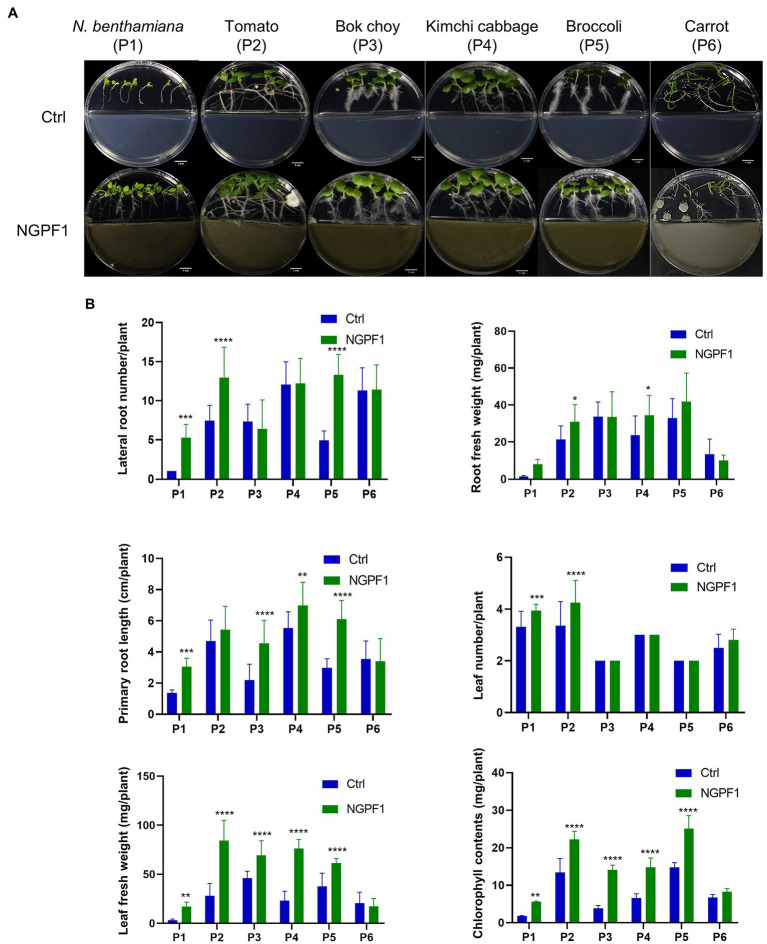
Effects of VOCs of *Cladosporium halotolerans* NGPF1 on plant growth enhancement *in vitro*. Growth of *N. benthamiana* (P1), tomato (P2), bok choy (P3), kimchi cabbage (P4), broccoli (P5), and carrot (P6) in a shared atmosphere with *Cladosporium halotolerans* NGPF1 **(A)** for 4days, except P1 and P2 for 10days. **(B)** The effects of VOCs on leaf fresh weight, root fresh weight, leaf number, lateral root number, root length, and chlorophyll contents were recorded after 4 days and 10 days exposure. Error bars indicate the standard deviation of the mean (*n*=18). Asterisks indicate a statistically significant difference between control (ctrl, no treatment) and NGPF1 treatment (two-way ANOVA, ^*^p<0.05, ^**^p<0.01, ^***^p<0.001, and ^****^p<0.0001).

To investigate the effects of *C. halotolerans* NGPF1 VOCs on growth of different plants, 1×10^8^ spores were inoculated on PDA medium in half of an I-plate (100×15mm) for 7days. Surface-sterilized seeds of bok choy (P3), kimchi cabbage (P4), broccoli (P5), and carrot (P6) were co-cultured onto MS medium on the other side of the I-plate at 25°C±1°C for 4days, while 6-day-old tomato (P1) and *N. benthamiana* (P2) seedlings were co-cultured for 10days (Figure 2A). This technique avoids physical contact between plants and the fungal strain. Plants exposed to *C. halotolerans* NGPF1 exhibited a significant increase in shoot and root biomass except for carrot (P6), and the VOC-treated plants were visually longer and had larger leaves compared with controls (Figure 2A). In addition, in the plants exposed to the VOCs of *C. halotolerans* NGPF1, the lateral root number was increased 1.0–7.0-fold compared with controls, while root fresh weight and root length were increased 0.75–6.18-fold and 0.96–2.07-fold, respectively, compared with controls. These differences constituted a significant increase of root fresh weight by the root length elongation and increased numbers of the lateral root. Although no significant difference was observed in leaf number between VOC treatment and control plants (1.00–1.18-fold), in all the species tested, except carrot, the leaf fresh weight (0.84–5.42-fold) and chlorophyll contents (1.21–3.66-fold) were significantly increased by exposure to the VOCs. The growth of carrot plants was not enhanced by exposure to the VOCs compared with control treatment, in any of the examined parameters, including lateral root number (1.0-fold), leaf number (1.12-fold), leaf fresh weight (0.84-fold), root fresh weight (0.75-fold), root length (0.96-fold), and chlorophyll contents (1.21-fold) (Figure 2B).

**Figure 3:**
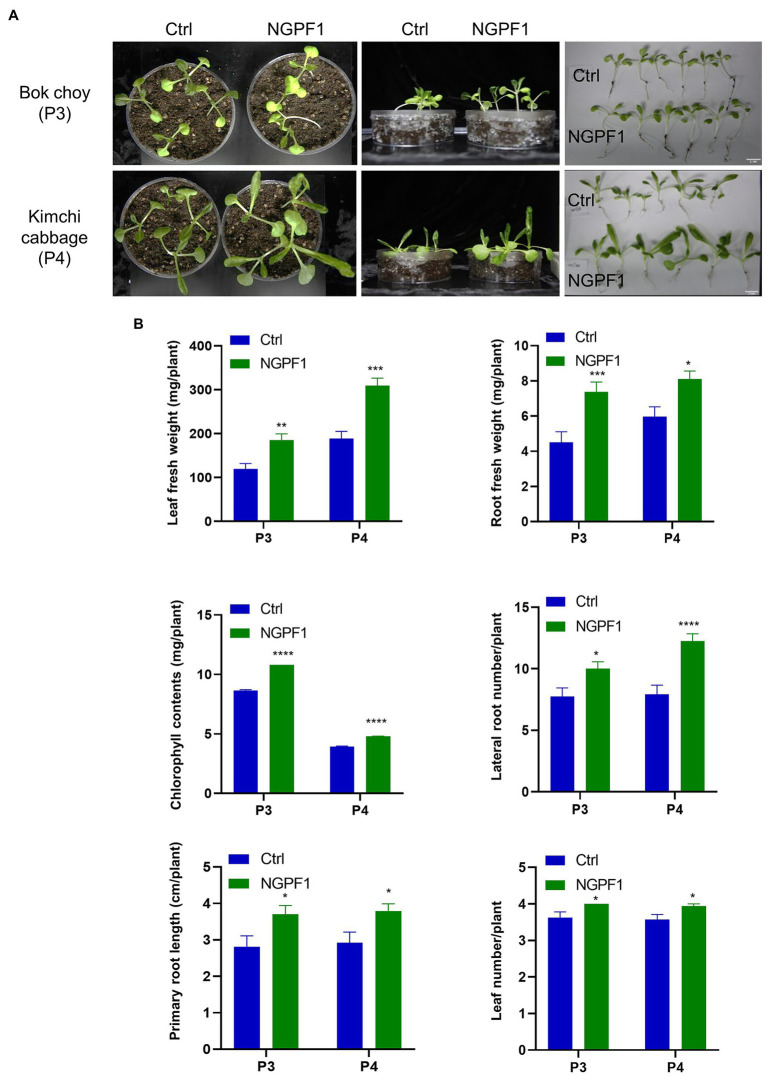
Effects of VOCs of *C. halotolerans* NGPF1 on plant growth enhancement in soils. **(A)** Growth promotion of bok choy and kimchi cabbage with VOCs of *C. halotolerans* NGPF1 in soils. **(B)** Quantification of leaf fresh weight, leaf number, chlorophyll contents, root fresh weight, lateral root number, and root length after exposure to fungal VOCs. Error bars indicate the standard deviation of the mean (*n*=15). Asterisks indicate a statistically significant difference between control (ctrl, no treatment) and NGPF1 treatment (two-way ANOVA, ^*^p<0.05, ^**^p<0.01, ^***^p<0.001, and ^****^p<0.0001). Experiments were repeated twice with similar results.

To further investigate if the VOCs had a PGP effect at a broader level, soil experiments were performed. Bok choy (P3) and kimchi cabbage (P4) were selected for the soil experiments assays because those two plants showed the greatest effect in response to VOCs in the I-plate assay. Surface-sterilized seeds were sown on the soil of petri dishes and a single plate of a 7-day-old culture of strain NGPF1 was placed next to the plant dish. This technique means that only the VOCs can contact plants, mimicking the natural conditions. The plants were exposed to VOCs of strain NGPF1 for 10days. Bok choy and kimchi cabbage plants exposed to VOCs had larger leaves and longer plant length compared with the control (Figure 3A). Growth of the plants in soil was significantly stimulated when exposed to strain NGPF1. The leaf and root fresh weight were increased 1.54- and 1.64-fold, respectively, for bok choy, and 1.64- and 1.35-fold, respectively, for kimchi cabbage. Furthermore, chlorophyll contents were increased by 1.25- and 1.22-fold for bok choy and kimchi cabbage, respectively (Figure 3B). These data proved that growth of the two plants, in terms of plant biomass and chlorophyll contents, was significantly enhanced by the VOCs (Figure 3B). Moreover, plant growth could be stimulated by VOCs of *C. halotolerans* NGPF1 in both the I-plate and soil conditions.

### Effects of Culture Media on PGP Activities

**Figure 4:**
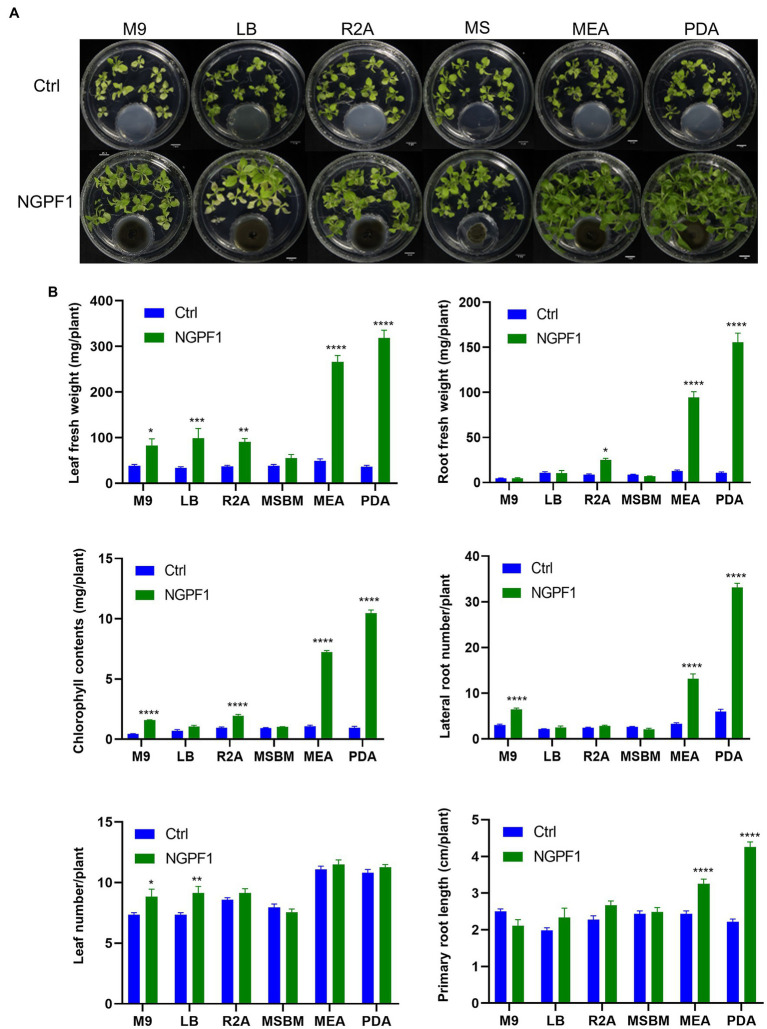
Degree of plant growth promotion mediated by VOCs of *C. halotolerans* NGPF1 on culture medium-dependent. **(A)** *Nicotiana benthamiana* growth on MS medium after co-cultivation with VOCs emitted by *C. halotolerans* NGPF1 grown on different culture media. **(B)** Quantification of leaf fresh weight, leaf number, chlorophyll contents, root fresh weight, lateral root number, and root length after exposure to fungal VOCs. Error bars indicate the standard deviation of the mean (*n*=18). Asterisks indicate a statistically significant difference between control (ctrl, no treatment) and NGPF1 treatment (two-way ANOVA, ^*^p<0.05, ^**^p<0.01, ^***^p<0.001, and ^****^p<0.0001). Experiments were repeated three times with similar results.

To investigate whether the PGP effects of *C. halotolerans* NGPF1 VOCs were dependent on the culture medium, 7-day-old *N. benthamiana* seedlings were co-cultivated on MS medium in 100×40mm culture dishes, while *C. halotolerans* NGPF1 was cultured in various different media, namely PDA, LBA, MEA, R2A, M9A, and MS, using 35×10mm culture dishes (Figure 4A). After 20days of exposure, the plant biomass of *N. benthamiana* seedlings was visibly significantly enhanced by the VOCs produced by *C. halotolerans* NGPF1 grown on PDA and MEA, with marked increases detected in the number of lateral roots, leaf fresh weight, root fresh weight, and total chlorophyll contents (Figure 4B). The VOCs produced by culture of the fungus on MEA and PDA media generated an increase of 5.36- and 8.75-fold, respectively, in leaf fresh weight, a 7.45- and 14.50-fold increase, respectively, in root fresh weight, a 6.77- and 11.16-fold increase, respectively, in chlorophyll contents, and an increase of 3.98- and 5.57-fold, respectively, in root numbers, but there were no significant differences in leaf numbers. These observations confirmed the PGP potential of VOCs of *C. halotolerans* NGPF1. However, growth of *N. benthamiana* seedlings exposed to *C. halotolerans* NGPF1 grown in M9 and MS media was not significantly different from the control (Figure 4B). This suggests that VOCs derived from *C. halotolerans* NGPF1 can play a vital role in growth promotion of *N. benthamiana*, but this ability is dependent on the culture medium of the fungal strain.

### Identification of VOCs Emitted by *C. halotolerans* NGPF1

From the results above, VOCs produced by *C. halotolerans* NGPF1 could stimulate plant growth in a culture medium-dependent manner, with the PGP activity only observed when the fungal strain was cultured in PDA and MEA media. Therefore, the VOCs emitted by *C. halotolerans* NGPF1 under these culture conditions were characterized using SPME coupled with GC–MS.

*C. halotolerans* NGPF1 was cultured in PD liquid medium at 25°C for 7days, while the strain was cultured in MS medium as a control. Following analysis by SPME coupled with GC–MS, the resulting mass spectra data of the volatile compounds were examined based on data in the NIST/EPA/NIH Mass Spectrum Library. Seven prominent volatile compounds were identified from *C. halotolerans* NGPF1 grown in a PD liquid medium (Supplementary Fig. S1A). Retention times of these compounds ranged from 4 to 7min, while molecular weights ranged from 44.03 to 86.07Da. Four of the compounds were identified as aldehydes (2-methyl-butanal, 3-methyl-butanal, acetaldehyde, 2-methyl-propanal), two were ketones (2,3-butanedione and acetone), and one compound was an acid (acetic acid). The acetaldehyde displayed the highest peak of the nine compounds detected on the VOCs spectrum emitted by *C. halotolerans* NGPF1. Twelve VOCs were identified from the control culture of *C. halotolerans* NGPF1 grown in MS medium (Table 1). Among all the volatile compounds from the two culture conditions examined, two of them—2-methyl-butanal and 3-methyl-butanal—were uniquely released by *C. halotolerans* NGPF1 in the PD culture medium. This implied that these two compounds may play a vital role in the PGP response induced by *C. halotolerans* NGPF1. Consequently, 2-methyl-butanal and 3-methyl-butanal were selected for further analysis.

**Table 1:** Identification of volatile organic compounds produced by *C. halotolerans* NGPF1 grown on two different media.

Compound no.	Peak assignment	CAS number	Molecular formula	Media	
				MS	PD
1	Acetaldehyde	000075-07-0	C_2_H_4_O	+	+
2	Acetone	000067-64-1	C_3_H_6_O	+	+
3	Propanal, 2-methyl-	000078-84-2	C_4_H_8_O	+	+
4	2,3-butanedione	000431-03-8	C_4_H_6_O_2_	+	+
5	Acetic acid	000078-93-3	C_4_H_8_O	+	+
6	2-butanone	000064-19-7	C_2_H_4_O_2_	+	−
7	2,3-butanediol	000513-85-9	C_4_H_10_O_2_	+	−
8	Heptane, 2,4-dimethyl-	002213-23-2	C_9_H_2_0	+	−
9	Furfural	000098-01-1	C_5_H_4_O_2_	+	−
10	Octane, 4-methyl-	002216-34-4	C_9_H_2_0	+	−
11	Benzaldehyde	000100-52-7	C_7_H_6_O	+	−
12	Decane	000124-18-5	C_10_H_22_	+	−
13	3-methyl-butanal	000590-86-3	C_5_H_10_O	−	+
14	2-methyl-butanal	000096-17-3	C_5_H_10_O	−	+

### Assessment of PGP Effect by Specific VOCs

**Figure 5:**
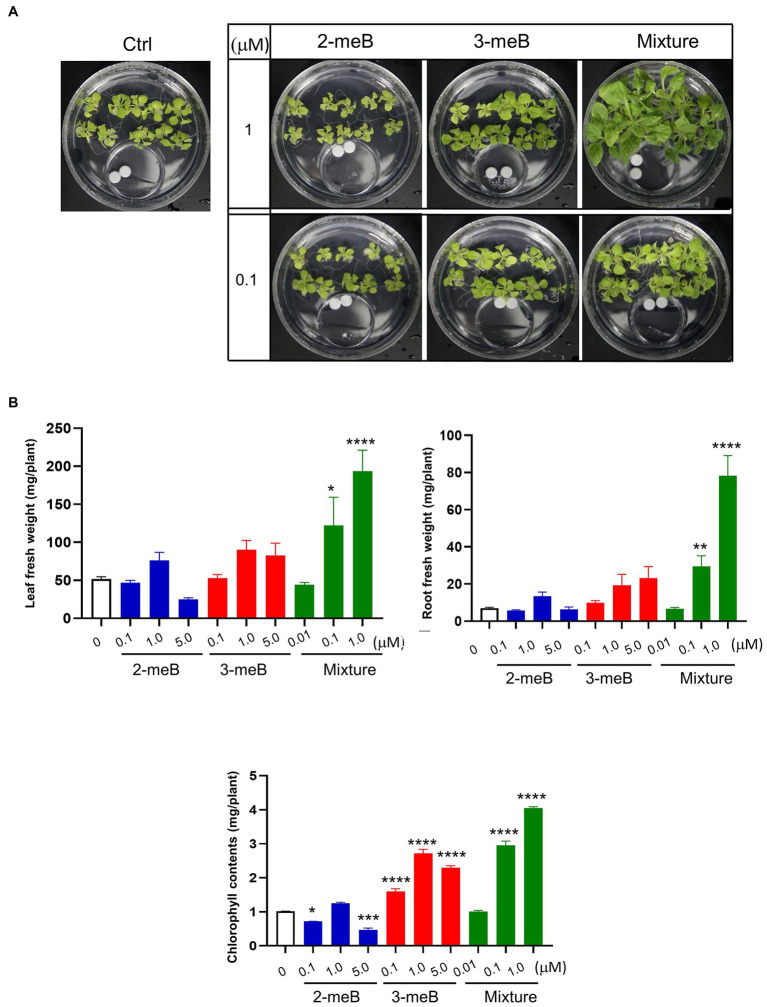
Effects of different concentrations of 2-methyl-butanal and 3-methyl-butanal on growth of *N. benthamiana*. **(A)** Phenotypic characteristics of the plant after co-cultivation with 2-methyl-butanal and 3-methyl-butanal. **(B)** 2-methyl-butanal and 3-methyl-butanal regulate leaf fresh weight, leaf number, chlorophyll contents, root fresh weight, lateral root number, and root length. Error bars indicate the standard deviation of the mean (*n*=18). Experiments were repeated three times with similar results. Asterisks indicate significant difference among no treatment and each chemical exposure in different concentrations (One–way ANOVA, ^*^p<0.05, ^**^p<0.01, ^***^p<0.001, and ^****^p<0.0001).

Different concentrations of 2-methyl-butanal and 3-methyl-butanal, which are only emitted from *C. halotolerans* NGPF1 during culture on PDA medium, were used to examine the effects of plant growth promotion. Seven-day-old *N. benthamiana* seedlings were grown in 100×40mm culture plates and co-cultured with 2-methyl-butanal (0.1μM, 1μM, and 5μM), 3-methyl-butanal (0.1μM, 1μM, and 5μM) or a blend of the two compounds (0.01μM, 0.1μM, and 1μM of each compound) applied on a filter paper disc in the 35×10mm culture dishes as described above. The plates were incubated for 6days, and visual inspection revealed that the blend of the two compound blends (1μM of 2-methyl-butanal and 1μM of 3-methyl-butanal) enhanced growth of *N. benthamiana* plants compared with control and other treatments (Figure 5A). The fresh root weight in the presence of the compound mixture was increased 11.25-fold, while the leaf fresh weight was increased 3.75-fold, and chlorophyll contents were increased 3.99-fold compared with the control (Figure 5B). With increasing concentrations of the compounds, the plant biomass increased and then decreased, indicating that high concentrations of the two VOCs could inhibit plant growth. In summary, these results provide strong evidence that the mixture of 2-methyl-butanal and 3-methyl-butanal emitted by *C. halotolerans* NGPF1 mediates plant growth.

### Expression of Genes Involved in Plant Growth Promotion

To investigate the possible mechanism underlying the promotion of plant growth by VOC mixtures and whether it involved phytohormone biosynthesis and metabolism, the relative expression level of phytohormone-associated genes was investigated *via* real-time PCR using primers specific for the genes encoding gibberellin (*GA20ox-1*), ethylene (*ACO1*), cytokinin (*SICKX1*), expansin (*Exp9*, *Exp18*), and auxin (*SIAA1*, *SIAA3*). Total mRNA was extracted from leaves exposed to *C. halotolerans* NGPF1 VOC mixtures following a time-course of 5, 7, 9, and 10days.

**Figure 6:**
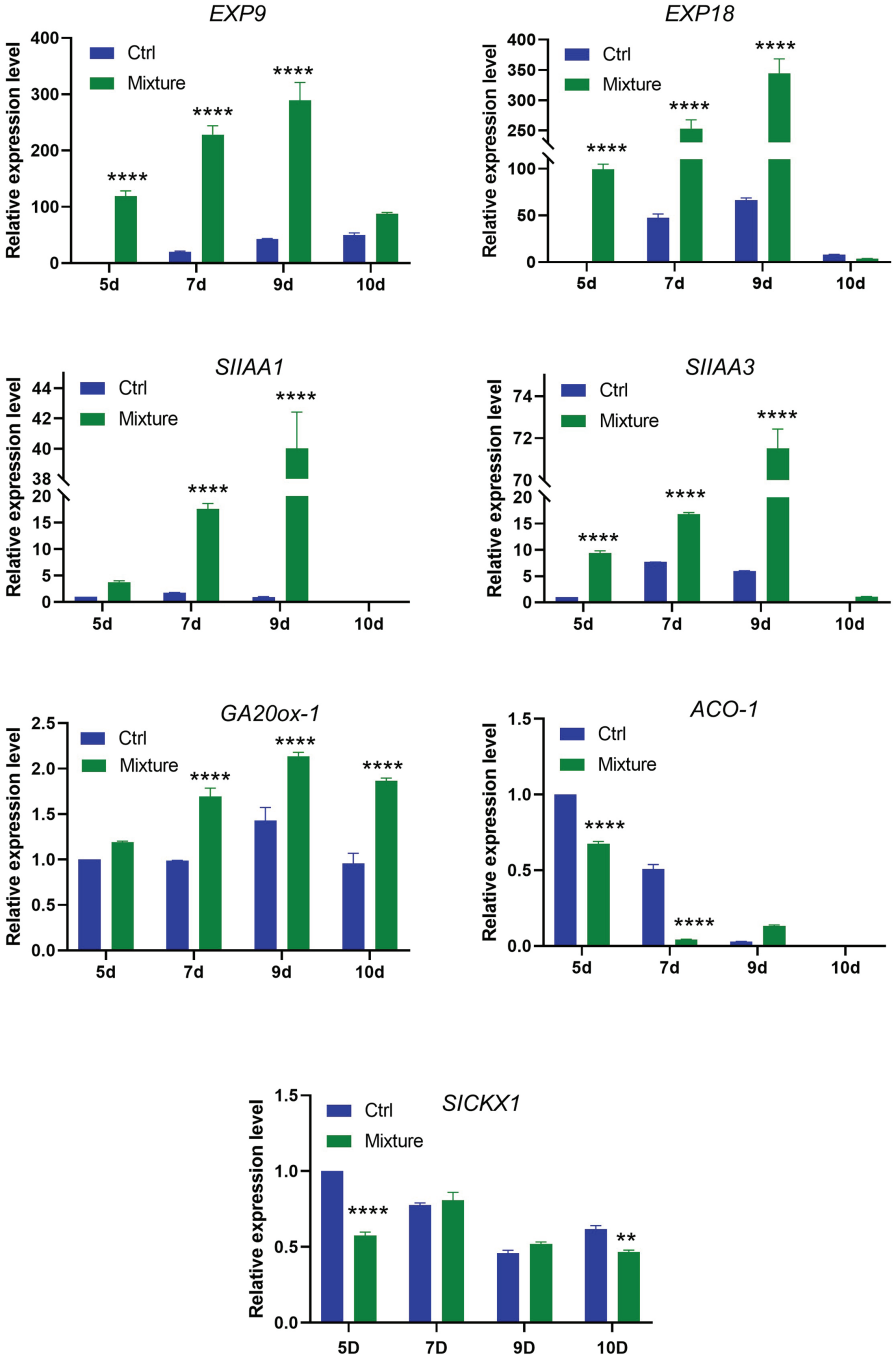
Expression profiles of genes involved in expansin, auxin, cytokinin, and gibberellin synthesis and metabolism following exposure to 2-methyl-butanal and 3-methyl-butanal. Plants were exposed to blends of 2-methyl-butanal and 3-methyl-butanal (1μM+1μM) for a time course of 5, 7, 9, and 10days. qRT-PCR was performed with SYBR and relative expression levels of genes related to expansin (*EXP9*, *EXP18*), auxin (*SIAA1*, *SIAA3*), gibberellin (*GA20ox-1*), ethylene (*ACO1*), cytokinin (*SICKX1*) biosynthesis and metabolism were determined. Asterisks indicate significant difference among no treatment (Ctrl) and exposure to two chemical mixtures (Mixture) in each time (two-way ANOVA, ^**^p<0.01, and ^****^p<0.0001).

Real-time PCR data revealed that relative expression of genes related to expansin (*Exp9*, *Exp18*), auxin (*SIAA1, SIAA3*), and gibberellin (*GA20ox-1*) was significantly higher in plants exposed to *C. halotolerans* NGPF1 VOC mixtures at 5–9days compared with the controls (Figure 6). The genes involved in the auxin and expansin pathways all exhibited a continuous increase in expression level with increasing time exposure to the VOC mixtures, and the highest expression level was detected on day 9. In contrast, the genes related to cytokinin (*SICKX1*) did not exhibit any increase in expression levels between control samples and leaves exposed to VOC mixtures at some time points. The gene related to ethylene (*ACO-1*) showed a marked downregulation in expression after exposure to the VOC mixtures (Figure 6). The gene *ACO-1* encodes 1-aminocyclopropane-1-carboxylic acid (ACC) oxidase, an enzyme that catalyzes a step in the ethylene biosynthesis process. These data strengthen support for the involvement of the auxin and expansin pathways in the PGP activity of VOC mixtures.

## Discussion

The genus *Cladosporium* is a well-known group of fungi that includes some indoor and outdoor molds. Many species of the genus *Cladosporium* are endophytes that live inside plants, and were found to promote plant growth rather than being pathogenic (Khalmuratova et al., 2020; Sun et al., 2020; Faddetta et al., 2021; Mao et al., 2021; Schmidt et al., 2021). However, some species of *Cladosporium*, including *C. fulvum* and *C. herbarum*, are phytopathogenic (Kaur and Manhas, 2014; Elgorban et al., 2017; Wang et al., 2018), strongly affecting plant growth and health. Exploration of the biological application of species of the genus *Cladosporium* is therefore necessary and worthwhile to prevent phytopathogen diseases as well as growth promotion of plants.

Fungal VOCs that have the ability to promote plant growth can avoid potential pathogenicity problems associated with whole organisms as direct contact is not required (Hamayun et al., 2010; Ghirardo et al., 2011; Paul and Park, 2013; Naznin et al., 2014; Li et al., 2018; Khalmuratova et al., 2020). It could be safer to only apply VOCs as bio-fungicides and growth stimulants to enhance the growth of plants. For example, *C. cladosporioides* CL-1 promoted tobacco seedling growth, improving the fresh weights and root development of the seedlings (Paul and Park, 2013), while *C. sphaerospermum* TC09 triggered growth promotion of tobacco seedlings and pepper plants, increasing fruit yield and early flowering (Li et al., 2018). In addition, *Cladosporium* sp. D-C-4 induced systemic resistance of *Arabidopsis thaliana* and reduced disease severity (Naznin et al., 2014). In the present study, a fungus isolated from contaminated medium of *N. benthamiana* seedlings was identified as *C. halotolerans* NGPF1 (Figure 1). This fungus promoted growth of some crop plants, including *N. benthamiana*, Micro-Tom tomato, bok choy, kimchi cabbage, and broccoli, in culture media and in soil tests. The measured plant biomass parameters of leaf fresh weight, root weight, root length, lateral root number, and chlorophyll contents all increased following exposure to *C. halotolerans* NGPF1 on the I-plate and in soil (Figure 2 and Figure 3). The inclusion of many crop plants in the current study, such as Micro-Tom tomato, bok choy, kimchi cabbage, and broccoli in addition to *N. benthamiana*, highlighted the potential benefits and opportunities to utilize VOCs of the fungus *C. halotolerans* NGPF1 widely in the agriculture industry.

There are 2.2 to 3.8 million species of fungi (Hawksworth and Lucking, 2017), and they are known to emit a large number of VOCs as mixtures of alcohols, monoterpenes, ketones, esters, small alkenes, sesquiterpenes, and derivatives (Morath et al., 2012; Inamdar et al., 2020). According to a recent tally (Li et al., 2018), 400 microorganisms from a total of 10,000 can produce more than 1,000 VOCs (Morath et al., 2012; Inamdar et al., 2020; Camarena-Pozos et al., 2021). Volatile profiles are influenced by numerous parameters such as environmental conditions like humidity, temperature, and pH, as well as the culture medium, culture time, species, and so on (Bitas et al., 2013; Lee et al., 2015; Maki et al., 2019; Rinnan et al., 2020). Due to variations in experimental design, data from different laboratories are frequently incomparable. In the present study, VOCs by *C. halotolerans* NGPF1 on different culture media were explored with seedlings of *N. benthamiana* using a modified plate-within-a-plate method (Figure 4). The plant growth response of *N. benthamiana* to VOCs emitted by *C. halotolerans* NGPF1 was dependent on the culture medium of the fungal species (Table 1; Supplementary Fig. S1).

Plant biomass was significantly improved through the production of VOCs on PDA and MEA media, but when *C. halotolerans* NGPF1 was cultured on M9, LB, R2A, and MS media, there were no significant increases in primary and lateral root length or lateral root density (Figure 4). This was supported by similar findings with *C. sphaerospermum* where a relatively higher level of plant growth was promoted when cultured on PDA medium (Li et al., 2018). However, in contrast with the study of Li et al. (2018), MS medium in the current study did not exhibit the highest stimulatory effect. In the present study, *C. halotolerans* NGPF1 grown on MS medium exhibited the least effect on PGP activity, and the plant growth effect was similar to that of Hutner’s medium. MS medium contains some vitamins and the same amount of sucrose as Hutner’s medium, which showed the lowest level of plant growth in the study by Li et al. (2018). When fungi are cultured in different media, some compounds are produced while others disappear. The VOC profiles, obtained by GC–MS, from *C. halotolerans* NGPF1 cultured in PD and MS media were compared and 12 different VOCs were identified from culture in MS media, while seven different compounds were identified following culture in PD medium. Among all the identified VOCs, acetaldehyde, acetoin (3-hydroxy-2-butanone), 2-methyl-propanal, 2,3-butanedione, and acetic acid were detected in both media. Seven unique compounds were produced following culture in MS medium, while two characteristic compounds (2-methyl-butanal and 3-methyl-butanal) were unique to PD medium culture.

Although numerous fungal VOC profiles have been collected and detected, the study of the function of different VOCs is still in the initial stages as additional data is needed. For example, 51 unique VOCs were detected from the fungus *Trichoderma*, but no functional studies were performed (Hung et al., 2013). Conversely, many compounds are inhibitory to plant growth or neutral, with only a few VOCs promoting plant growth. For example, Lee and co-workers isolated 141 unique compounds from nine strains of *Trichoderma*, but only 18 VOCs induced plant growth (Lee et al., 2016). In the current study, acetone, 2-methyl-propanol, and 2,3-butanedione were found in both media. 2,3-butanedione was detected in many microbiomes, including *Bacillus*, *Rhodococcus*, *Escherichia*, *Serratia*, *Paenibacillus*, *Lactobacillus*, and it was shown to promote *Arabidopsis* growth (Ryu et al., 2003; Ryu et al., 2004; Wang et al., 2013; Zhang et al., 2014; Yu et al., 2015; Rath et al., 2018; Zheng et al., 2020). In addition, 2-methyl-propanol also improved the biomass of *Arabidopsis* (Ryu et al., 2004; Naznin et al., 2013).

In the current study, different plant responses were observed following exposure to the VOCs emitted from PDA and MS media. Therefore, the two compounds that were uniquely produced in PDA medium (2-methyl-butanal and 3-methyl-butanal) were selected for further analysis and characterization of their effects on plant growth. As expected, a mixture of the two compounds stimulated plant growth and development. Although the single compounds partially increased plant biomass, root biomass, and chlorophyll contents, significant improvements in growth were only found with the mixture of 2-methyl-butanal (1μM) and 3-methyl-butanal (1μM). These two compounds are commonly found in other PGP fungi, including *Trichoderma atroviride* (GJS 01–209) (Lee et al., 2015), *Bacillus amyloliquefaciens*, and *Aspergillus oryzae* (Seo et al., 2018; Sun et al., 2018). At the same time, 3-methyl-butanal was found as the predominant volatile compound in *Phoma* sp., a plant growth-promoting fungus, and promoted tobacco growth *in vitro* (Naznin et al., 2013). Interestingly, blends of 3-methyl-butanal with other compounds had a more significant effect on plant growth compared with 3-methyl-butanal alone, and the ratio of each compound played an important role in promoting plant growth (Naznin et al., 2013). Dose-dependent regulation of plant growth by VOCs was also observed in the current study.

Some VOCs were previously reported to influence plant growth, but studies on the interaction between VOCs and plants are limited and the exact mechanisms of VOCs on phytohormones, and expression of related genes or signaling pathways have yet to be elucidated. PGP activity predominantly occurs through regulation of plant hormones and cell wall extension, like gibberellins, cytokinins, ethylene, auxin, and expansin, which are important modulators of plant growth. In the current study, blends of 2-methyl-butanal and 3-methyl-butanal stimulated plant growth by regulating phytohormones, markedly increasing the expression level of auxin and expansin genes, which increased the biomass of the plants. Auxins are a class of plant hormones that have a cardinal role in the coordination of many growth processes in plant life cycles and are essential for plant body development. In this study, auxin-related genes were highly increased when exposed to the blend of VOCs. This is supported by findings with VOCs from the bacteria *Bacillus subtills*, which induced auxin-related gene expression (Zhang et al., 2007; Tahir et al., 2017). *Fusarium oxysporum* VOCs also mediated plant growth *via* an auxin signaling pathway (Bitas et al., 2015). VOCs emitted from *Trichoderma atroviride* regulate the primary root vasculature of *Arabidopsis via* auxin signaling pathways (Garnica-Vergara et al., 2016). Expansin-related genes were reported to be upregulated in *Arabidopsis* (*EXPB1*, *EXPB3*, *EXP4*, and *EXP5*) (Zhang et al., 2007), in *Nicotiana* (*EXP1*, *EXP2*, and *EXP6*) (Xie et al., 2014), and in *Lactuca sativa* (*EXPA5*) (Minerdi et al., 2009) following exposure to VOCs. Similarly, the current study showed a higher expression of expansin genes (*EXP9*, *EXP18*), leading to cell expansion.

In conclusion, the fungus *C. halotolerans* NGPF1 emitted VOCs to stimulate plant growth in culture media and soil experiments, and the VOCs profile was medium-specific. Of all the VOCs identified, 2-methyl-butanal and 3-methyl-butanal were instrumental in promoting plant growth. Expression levels of genes related to auxin, expansion, gibberellin, cytokinin, and ethylene suggest the VOCs stimulate plant growth *via* auxin hormone and expansin signal transduction pathways. Consequently, we hypothesize that *C. halotolerans* NGPF1 emits 2-methyl-butanal and 3-methyl-butanal that contribute to plant growth promotion and act as signaling molecules *via* an auxin-related pathway. The findings from this study have significant implications in the agricultural application of VOCs for plant growth.

## Data Availability Statement

The datasets presented in this study can be found in online repositories. The names of the repository/repositories and accession number(s) can be found in the article/Supplementary Material.

## Author Contributions

JL and SM contributed to the conception of the project, supervised the project, and edited the manuscript. LJ and MHL designed and carried out the experiments. LJ and JL curated data, wrote the original manuscript, and revised the manuscript. CK, SK, and PK edited the manuscript. All authors have read and agreed to the published version of the manuscript.

## Funding

This work was supported by Korea Institute of Planning and Evaluation for Technology in Food, Agriculture and Forestry (IPET) through the Agricultural Machinery/Equipment Localization Technology Development Program, funded by Ministry of Agriculture, Food and Rural Affairs (MAFRA) (321057051HD020), and by the KRIBB research initiative program (KGM5282122).

## Conflict of Interest

The authors declare that the research was conducted in the absence of any commercial or financial relationships that could be construed as a potential conflict of interest.

## Publisher’s Note

All claims expressed in this article are solely those of the authors and do not necessarily represent those of their affiliated organizations, or those of the publisher, the editors and the reviewers. Any product that may be evaluated in this article, or claim that may be made by its manufacturer, is not guaranteed or endorsed by the publisher.
