# Supplementary material for: Plant Growth Promotion by Two Volatile Organic Compounds Emitted From the Fungus Cladosporium halotolerans NGPF1
Source: Front Plant Sci. 2021 Dec 3;12:794349. doi: 10.3389/fpls.2021.794349 (PMC8678569; doi:10.3389/fpls.2021.794349)
Supplement: Supplementary file 1 [file Data_Sheet_1.PDF]

Supplementary Material

A

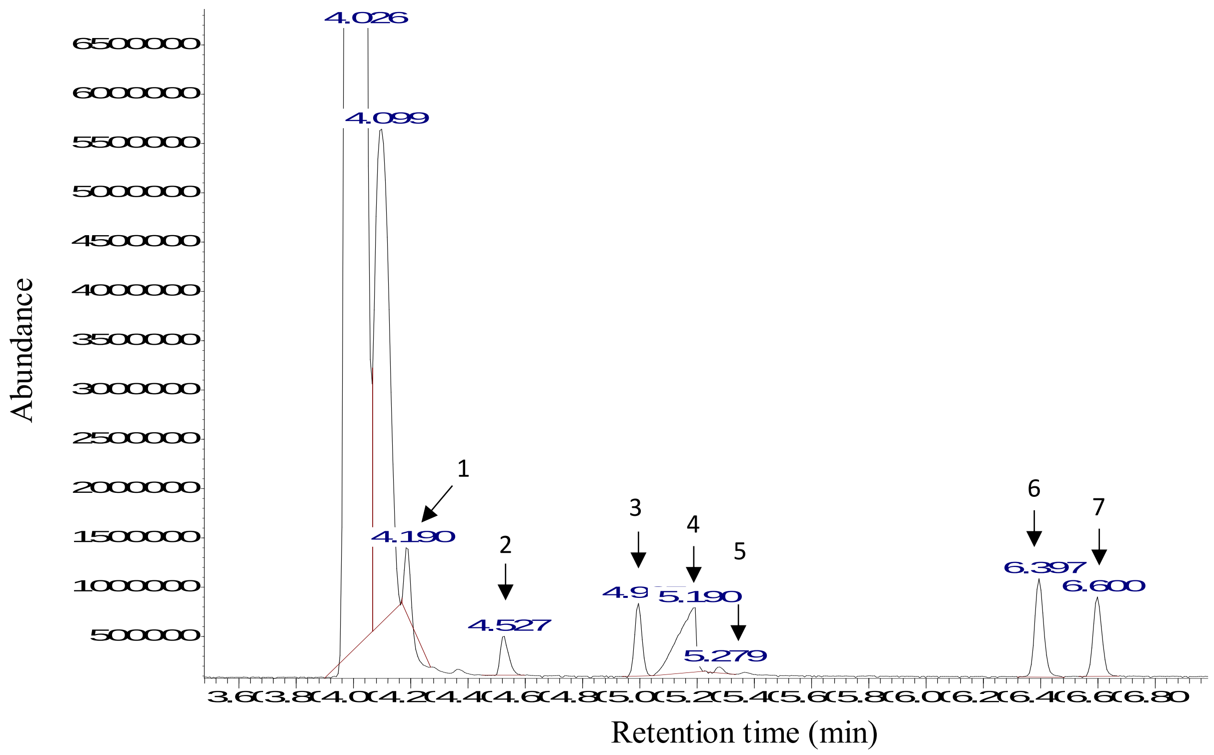

B

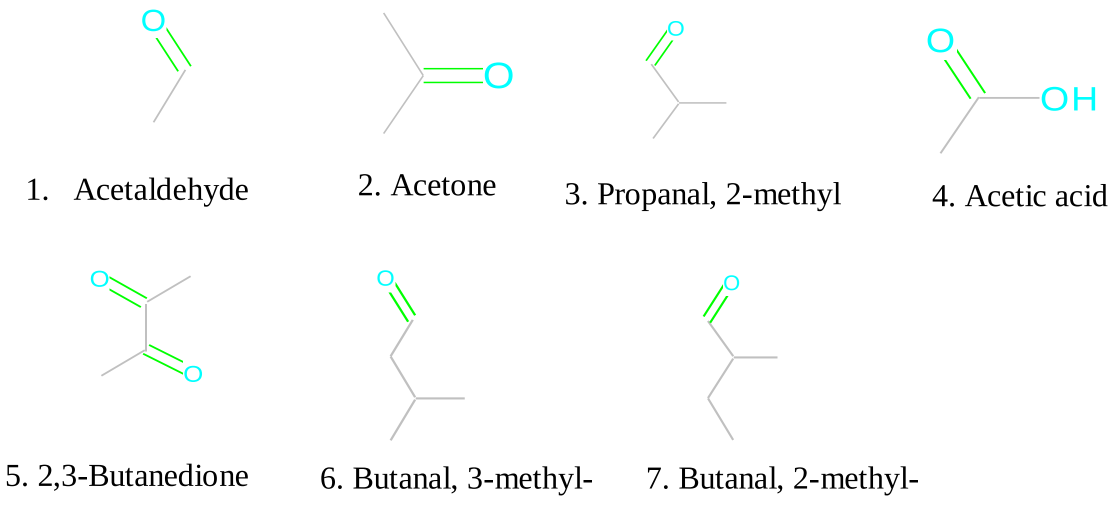

Fig. S1. Identification of volatile organic compounds (VOCs) emitted by *Cladosporium halotolerans* NGPF1. (A) Analysis of VOCs derived from *C. halotolerans* NGPF1 was performed using an HS-GC/MS system. The numbers of the peaks indicate the individual chemical compounds identified during the analysis. (B) Identification of VOC structures emitted by *C. halotolerans* strain NGPF1 by HS-GC/MS.
